# Single-cell analysis reveals ADGRL4+ renal tubule cells as a highly aggressive cell type in clear cell renal cell carcinoma

**DOI:** 10.1038/s41598-024-52928-1

**Published:** 2024-01-29

**Authors:** Zehua Wang, Zhongxiao Zhang

**Affiliations:** 1 Department of Urology, Qilu Hospital of Shandong University, Jinan, 250012 China; 2 Department of Urology, Qilu Hospital (Qingdao), Shandong University, Qingdao, 266035 China

**Keywords:** Urogenital diseases, Cancer therapy, Urological cancer

## Abstract

Clear cell renal cell carcinoma (ccRCC) is a highly heterogeneous cancer that poses great challenge to clinical treatment and prognostic prediction. Characterizing the cellular landscape of ccRCC in a single-cell dimension can help better understand the tumor heterogeneity and molecular mechanisms of ccRCC. This study analyzed single-cell profiles in ccRCC samples and para-tumor samples from Gene Expression Omnibus and identified a highly heterogeneous subcluster of renal tubule cells. Single-cell regulatory network inference and clustering analyses and cell communication analysis were performed to develop transcription factor-target gene regulatory networks and cell–cell interactions. Additionally, the distribution and prognostic risk of renal tubule cells from spatial transcriptome data (GSM6415706) and The Cancer Genome Atlas-Kidney Clear Cell Carcinoma data were analyzed. A total of 10 cell types were identified in ccRCC and para-tumor samples. The ccRCC renal tubule cells showed a high expression of the oncogene nicotinamide *N*-methyltransferase and a significantly high degree of tumor heterogeneity. We further identified 6 cell subclusters with specific expression of BEX2, PTHLH, SFRP2, KLRB1, ADGRL4, and HGF from the ccRCC renal tubule cells. ADGRL4+ renal tubule cells had highly metastatic and angiogenesis-inducing characteristics, with more ADGRL4+ renal tubule cells indicating a worse survival. ADGRL4+ renal tubule cells regulated the metastasis of other renal tubule cells through metastasis-related receptor-ligand communication. We also found that ADGRL4+ renal tubule cells clustered around the glomeruli but the rest of the renal tubule cell subclusters rarely localized in ccRCC tissues. ETS1 and ELK3 -dominant GRNs were remarkably activated in ADGRL4+ renal tubule cells, functionally, knockdown of ELK3 in A498 significantly disturbedaffected the cell migration and invasion. ADGRL4+ renal tubule cells, which were highly metastatic and invasive, might be an essential cell subcluster for ccRCC, and ADGRL4 could be used a novel therapeutic target.

## Introduction

Renal cancer is the most common cancer in the urinary tract and its incidence and mortality are on the rise, leading to approximately nearly 180,000 in 2020^1–3^. Clear cell renal cell carcinoma (ccRCC), the most common pathologic type of renal cancer, accounts for about 75% of all patients with renal cancer^4,5^. Anti-PD-L1 and CTLA-4 drugs have made some advances in treating ccRCC as a result of an increasing use of immune checkpoint blockade (ICB) therapy^6^. However, ccRCC is highly heterogeneous and complex in genetic predisposition^7,8^. Drug trials showed that more than 50% of ccRCC patients are insensitive to ICB therapy, and even patients initially benefiting from taking therapies will develop secondary resistance^9,10^. Therefore, mortality in advanced ccRCC continues to increase^11^.

Tumor heterogeneity refers to the phenomenon that tumor cells have different directions of mutation at the individual level due to environmental or genetic factors^12^. Although the genetic features and molecular mechanisms of ccRCC have been described^13^, previous study does not adequately explain tumor heterogeneity in ccRCC. Single-cell RNA sequencing (scRNA-seq) could depict immune cell landscape in the ccRCC immune microenvironment (TME)^14,15^. A comprehensive understanding of the immune cell landscape in the TME could help expand the application of ICB therapies to more patients. However, few studies probe into the heterogeneity and direction of mutation of ccRCC tumor cells, and we also face a lack of critical cell subpopulations that promote pathologic staging of ccRCC. Revealing the heterogeneous cell clusters in ccRCC and mining biomarkers critically involved in the molecular mechanisms might be helpful for improving the current survival status of ccRCC.

This study analyzed the cell profiles of ccRCC and para-tumor tissues to reveal critical and tumor invasion-promoting cell subclusters for the cancer. Combining spatial transcriptomic data and survival data, the impact of cell subclusters on the aggressiveness and survival of ccRCC was comprehensively studied.

## Materials and methods

### Data sources

In this study, scRNA-seq data of 12 samples were obtained, including 7 primary ccRCC samples and 5 para-tumor samples. The matrix data (GSE156632) were sourced from the Gene Expression Omnibus (GEO, https://www.ncbi.nlm.nih.gov/geo/) database. Sequencing data for the ccRCC project were acquired from the University of Cingifornia Sisha Cruz (UCSC)-Xena (http://xena.ucsc.edu/). The Cancer Genome Atlas (TCGA) Kidney Clear Cell Carcinoma (KIRC) (FPKM format data) was selected from the "TCGA Hub". After excluding the data of surgical effect on prognostic assessment, samples with survival time > 90 days (N = 501) were included for further analysis. In addition, GSM6415706 cohort from a spatial transcriptome sequencing project was collected from GEO. The tissue type was ccRCC samples (Stage III) and the patients without any treatment.

### Analysis of GSE156632 data

The data matrix of the samples in GSE156632 was using through the Seurat code package (4.3.0)^16^ installed in R software (3.6.0) for data filtering process under the filtering criteria that the number of expressed genes in a cell was between 200 and 6000 and mitochondria in the cell was < 10% for several days. The SCTransform function was run for data normalization. The RunPCA() function was run for principal component analysis (PCA). The harmony package^17^ in R was used to remove batch effects in samples from different sources. The processed cells were subjected to Uniform Manifold Approximation and Projection (UMAP) dimensionality reduction analysis, where the top 30 principal components were plotted into K-Nearest Neighbor (KNN) plots according to Euclidean distances. The FindCluster function in the Seurat package was used to perform cell clustering analysis to identify cell types in para-tumor and ccRCC samples. For all cells, resolution was set to 0.1 and for renal tubule cells, resolution was set to 0.3. The marker genes for the cells were sourced from the CellMarker database (http://biocc.hrbmu.edu.cn/CellMarker/ or http://bio-bigdata.hrbmu.edu.cn/CellMarker/)^18^ and the study by Hu et al.^19^ that identified subclusters of cells based on the expression levels of the marker genes.

### Identification of differentially expressed genes (DEGs) and functional annotation between cell clusters

DEGs (avg_log fold change (FC) > 0.25 and *p*_val_adj < 0.05) between cell subclusters were initially identified using the FindAllMarkers function in the Seurat package and then uploaded to the DAVID database (https://david.ncifcrf.gov/)^20^ for Gene Ontology (GO) function annotation analysis to show the GO_Biological Process (BP) (*p* value < 0.05).

### Single-cell regulatory network inference and clustering analysis

Single-cell regulatory network inference and clustering (SCENIC) analysis was performed based on the heterogeneous cell subclusters in ccRCC and para-tumor tissues to develop gene regulatory networks (GRNs) governed by transcription factors (TFs)^21,22^. The GENIE3 package^21,23^, the RcisTarget package, and the AUCell package^21^ were installed in R. All the genes in cell subclusters were trained in the GENIE3 package and used to develop a random forest model for selecting target genes for IFs. False-positive indirectly targeted by IFs binding motifs and candidate IFs were removed by the RcisTarget package, while only IFs-targets genes showing a direct relationship (regulons) were retained. The AUCell package was ran to assess the activity of regulons activity to determine whether the regulons were in an active or inactive state.

### Cell communication analysis

For the interaction density and strength of cell-to-cell ligand-receptor interactions between cell subclusters, cell communication analysis was conducted by running the CellChat package in R^24^ with the interaction types of "Cell–Cell Contact" and "Secreted Signaling". The interaction strength was shown as the ligand-receptor interaction bubbles.

### Spatial transcriptomics data analysis

The Load10X_Spatial function in the Seurat package was run to read the spatial transcriptome data in GSM6415706, and the SCTransform function was executed to normalize the sequencing data in the spot. The SpatialFeaturePlot function was used to show the expression of the genes of interest in the ccRCC tissue and mapped them to the tissue sections in GSM6415706. The FindTransferAnchors and TransferData functions in the Seurat package were used to determine the proportion of renal tubule cell subclusters.

### Survival analysis

Survival analysis was conducted for evaluating the impact of the abundance of each identified renal tubule cell subcluster on patients’ prognosis. The AverageExpression function in the Seurat package was used to assess the average expression values of genes in the renal tubule cell subcluster. In this process, the parameters of assays = "SCT" and slot = "data" were selected to determine the DEGs. Using the Cell-type Identification By Estimating Relative Subsets of RNA Transcripts (CIBERSORT) algorithm^25^, the proportion of renal tubule cells subclusters in each bulk tissue of TCGA-KIRC was assessed based on the average expression values of DEGs in the renal tubule cell subclusters. Based on the median value, samples in TCGA-KIRC were categorized into high- and low- abundance groups, among which prognostic differences were assessed by Kaplan–Meier (K-M) survival analysis with log-rank test (*p* < 0.05).

### Acquisition and culture of cells

Human renal cortical proximal tubular epithelial cell line HK-2 and human renal carcinoma cell line A489 were purchased from the American Type Culture Collection (ATCC). The cells were cultured according to the storage instructions and culture procedures on the ATCC website.

### Quantitative real-time reverse-transcription PCR (qRT-PCR)

Total RNA was isolated by cell lysis with TRIzol (Invitrogen), and cDNA was synthesized using SuperScript™ IV First-Strand Synthesis System (Invitroge). The mRNA levels were quantified using Hieff® qPCR SYBR Green Master Mix with GAPDH as a reference gene. The primer sequences were: ELK3-Forward Primer: GAGAGTGCAATCACGCTGTG, ELK3-Reverse Primer: GTTCGAGGTCCAGCAGATCAA;

ETS1- Forward Primer: GATAGTTGTGATCGCCTCACC, ETS1-Reverse Primer: GTCCTCTGAGTCGAAGCTGTC;

GAPDH- Forward Primer: 5′-CTGGGCTACACTGAGCACC-3′, GAPDH-Reverse Primer: 5′- AAGTGGTCGTTGAGGGCAATG-3′.

### Transfection of Si-ELK3 and Transwell assay

The siRNA for ELK3 and negative control siRNA were constructed by GenePharma (Shanghai, China) and transfected using Lipofectamine RNAiMAX (Invitrogen). Migration assay was conducted in 6.5 nm Transwell chambers with 8-µm pores (Corning, NY, USA). A498 cells were seeded in the upper chamber of the Transwell, and DMEM supplemented with 10% FBS was added to the lower chamber. After 24 h of incubation, the number of cells entering the lower chamber was stained and counted. Cell invasion assay was carried out according to the steps of coating basement membrane—hydration basement membrane—preparation of cell suspension—seeding cells to the upper chamber—cell fixation—cell staining—sealing—cell observation and counting.

### Statistical tests

All statistical analyses were conducted in the R language (version 3.6.0). Wilcoxon rank-sum test was used to analyze the difference between the two groups of continuous variables. Survival analysis differences were assessed by log-rank test. Sample screening in TCGA-KIRC was performed using SangerBox 3.0 (http://sangerbox.com/login.html)^26^. *P* < 0.05 was considered statistically significant for all statistical tests.

## Results

### Single-cell profiles in ccRCC and para-tumor tissues

**Figure 1:**
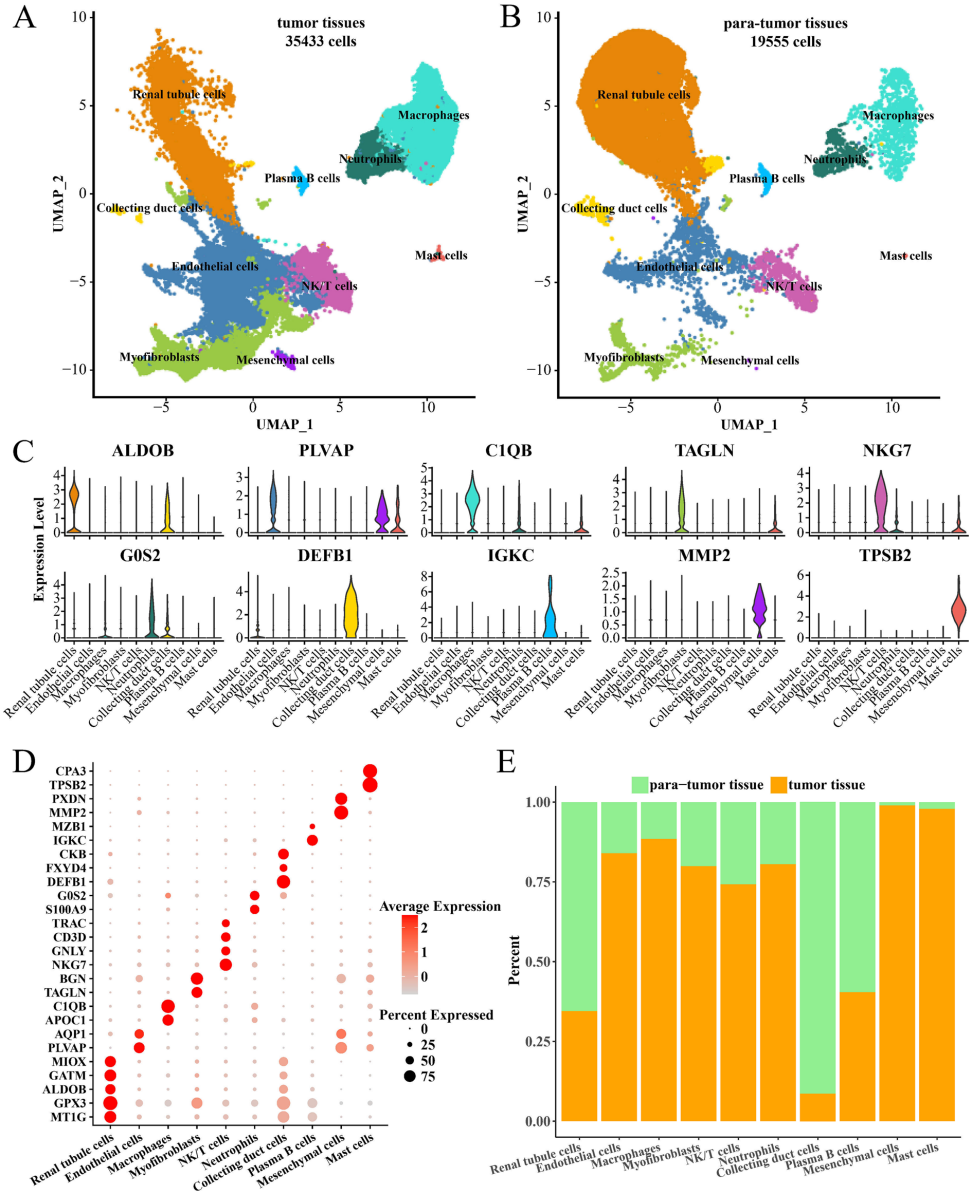
Single cell profiles in ccRCC and para-tumor tissues. (**A**) Distribution graph of 10 cell types clustered in CcRCC tissues. (**B**) Distribution graph of 10 cell types clustered in para-tumor tissues. Each cell type is labeled with a specific color. (**C**) Expression violin graph of marker genes in 10 cell types. (**D**) Expression bubble plot of marker genes in 10 cell types. The darker the red color of the bubble, the higher the average expression level of the gene. (**E**) Histogram of the percentage of 10 cell types in ccRCC and para-tumor tissues. Green represents paracancerous tissue and orange represents tumor tissue.

After processing the cell data in GSE156632, 54,988 cell data met the criteria for scRNA-seq analysis (Supplementary Fig. 1A). A total of 10 cell types (renal tubule cells, endothelial cells, macrophages, myofibroblasts, NK/T cells, neutrophils, collecting duct cells, plasma B cells, mesenchymal cells, mast cells) in ccRCC and para-tumor tissues were determined, with 35,433 cells in ccRCC and 19,555 cells in para-tumor tissues (Fig. 1A–B, Supplementary Fig. 1B–C). The expression status of some marker genes in the 10 cell types was shown in Fig. 1C and D. We found a high proportion of renal tubule cells, collecting duct cells, and plasma B cells in para-tumor tissues. Endothelial cells, macrophages, myofibroblasts, NK/T cells, neutrophils, mesenchymal cells, and mast cells were overrepresented in ccRCC tissues (Fig. 1E, Supplementary Fig. 1D). In addition, according to the oncogene Nicotinamide N-Methyltransferase (NNMT) in ccRCC, we found that renal tubule cells, endothelial cells in ccRCC tissues high-expressed NNMT, while the two types of cells in para-tumor tissues barely expressed NNMT (Supplementary Fig. 2). Our findings indicated that renal tubule cells, endothelial cells in ccRCC were cancerous cells, while those in para-tumor tissues might be non-cancerous cells.

### Renal tubule cells showed heterogeneity in ccRCC tissues

**Figure 2:**
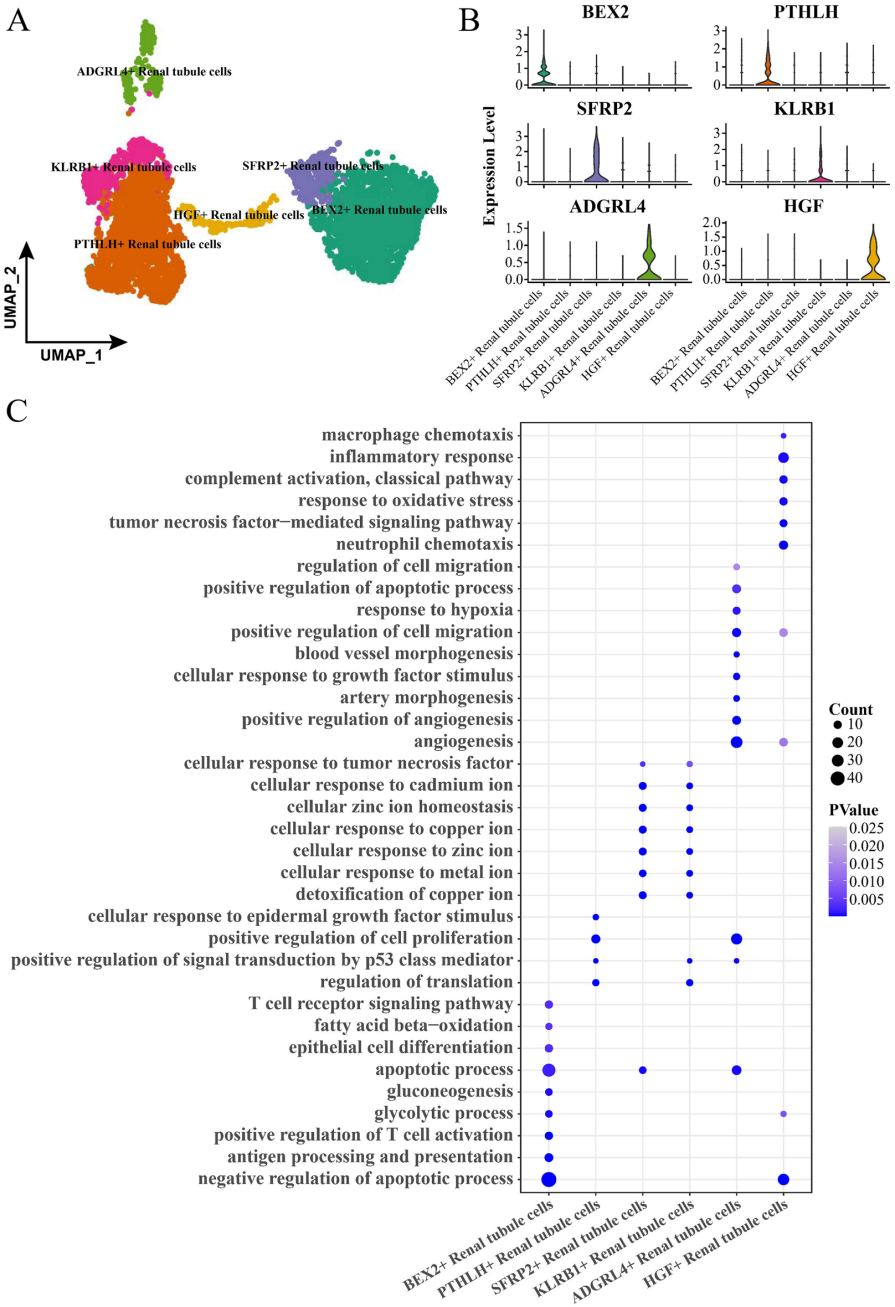
Cell landscape of 6 subclusters of renal tubule cells. (**A**) UMAP diagram for 6 subclusters of renal tubule cells in ccRCC tissues. (**B**) Expression violins of marker genes in the 6 Renal tubule cell subclusters. (**C**) Biological processes involved in the 6 Renal tubule cell subclusters. The heavier the blue color of the dot, the higher the degree of significance.

Pathologic studies confirmed that ccRCC is an adenocarcinoma originating from renal tubule cells^27^. Our findings also supported that all of the renal tubule cells in ccRCC tissues high-expressed the oncogene NNMT, therefore subsequent studies were performed with a focus on the heterogeneity of the renal tubule cells and the implications of biological functions. Renal tubule cells in ccRCC tissues and related data were extracted and subjected to UMAP dimensionality reduction clustering analysis and annotation. We identified six cell subclusters with specific high-expressed genes, namely, BEX2 (BEX2 + renal tubule cells), PTHLH (PTHLH + renal tubule cells), SFRP2 (SFRP2+ renal tubule cells), KLRB1 (KLRB+ renal tubule cells), ADGRL4 (ADGRL4+ renal tubule cells), HGF (HGF + renal tubule cells) (Fig. 2A–B). BEX2 + renal tubule cells were involved in the negative regulation of apoptotic process, antigen processing and presentation, positive regulation of T cell activation, glycolytic process, gluconeogenesis, apoptotic process, epithelial cell differentiation, fatty acid beta-oxidation, T cell receptor signaling pathway. PTHLH + renal tubule cells were involved in regulation of translation, positive regulation of signal transduction by p53 class mediator, positive regulation of cell proliferation, and cellular response to epidermal growth factor stimulus. SFRP2+ renal tubule cells and KLRB+ renal tubule cells were involved in some of the same biological processes, including detoxification of copper ion, cellular response to metal ion, cellular response to zinc ion, cellular response to zinc ion, cellular response to copper ion, cellular zinc ion homeostasis, cellular response to cadmium ion, cellular response to tumor necrosis factor. However, there were also some differences. SFRP2+ renal tubule cells were involved in the apoptotic process. KLRB+ renal tubule cells were involved in regulation of translation, positive regulation of signal transduction by p53 class mediator. ADGRL4+ renal tubule cells were involved in angiogenesis, positive regulation of angiogenesis, artery morphogenesis, cellular response to growth factor stimulus, blood vessel morphogenesis, positive regulation of cell migration, response to hypoxia, positive regulation of apoptotic process. HGF + renal tubule cells were involved in neutrophil chemotaxis, tumor necrosis factor-mediated signaling pathway, response to oxidative stress, complement activation, classical pathway, inflammatory response, macrophage chemotaxis (Fig. 2C).

### ADGRL4+ renal tubule cells regulated angiogenesis in ccRCC

**Figure 3:**
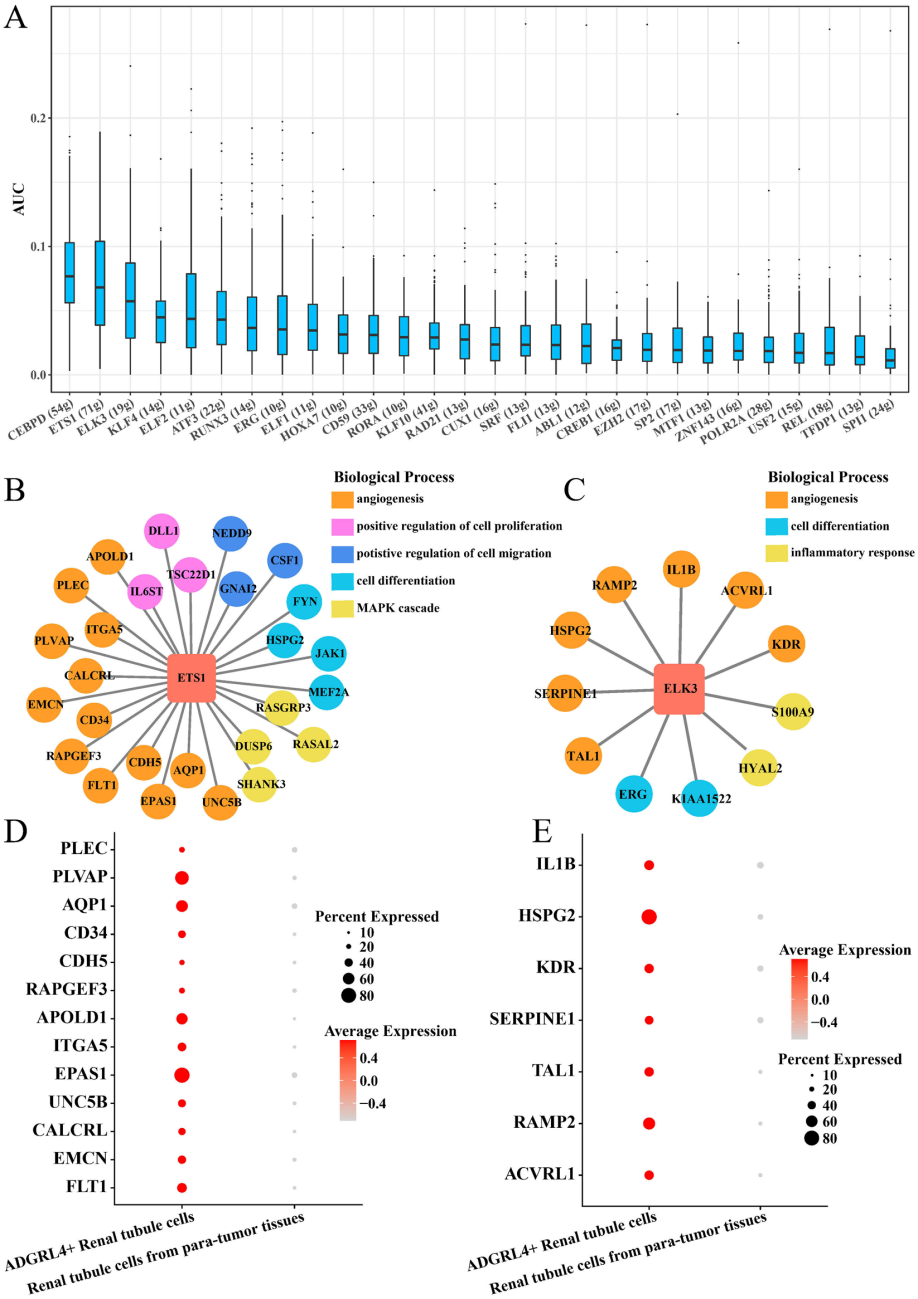
ADGRL4+ renal tubule cells regulate angiogenesis in ccRCC. (**A**) AUC score of regulons in ADGRL4+ renal tubule cells. (**B**) Biological processes involved in ETS1-interacting GRNs. Each biological pathway is represented by a color block in a specific color. (**C**) Biological processes involved in ELK3-interacting GRNs. (**D**) Expression levels of ETS1 target genes in ccRCC and para-tumor-derived renal tubule cells. (**E**) Expression levels of target genes of ELK3 in ccRCC and para-tumor-derived renal tubule cells.

Previous studies found that ADGRL4+ renal tubule cells were related to angiogenesis, positive regulation of angiogenesis, and blood vessel morphogenesis. Angiogenesis is closely involved in the invasive migration of cancer cells and contributes to cancer progression. In this study, we discussed the Ifs network of ADGRL4+ renal tubule cells associated with angiogenesis. SCENIC analysis identified 28 Ifs that directly acted as target genes (Fig. 3A). ETS1-interacting GRNs were significantly enriched in angiogenesis, positive regulation of cell proliferation, positive regulation of cell migration, cell differentiation, and MAPK cascade (Fig. 3B). ELK3-interacting GRNs were enriched to angiogenesis, cell differentiation, inflammatory response (Fig. 3C). Furthermore, we found that ETS1- and ELK3-interacting target genes were all up-regulated in ADGRL4+ renal tubule cells, and none of them were expressed in para-tumor-derived renal tubule cells (Fig. 3D–E).

### ADGRL4+ renal tubule cells might be an inducer of cell proliferation in ccRCC

**Figure 4:**
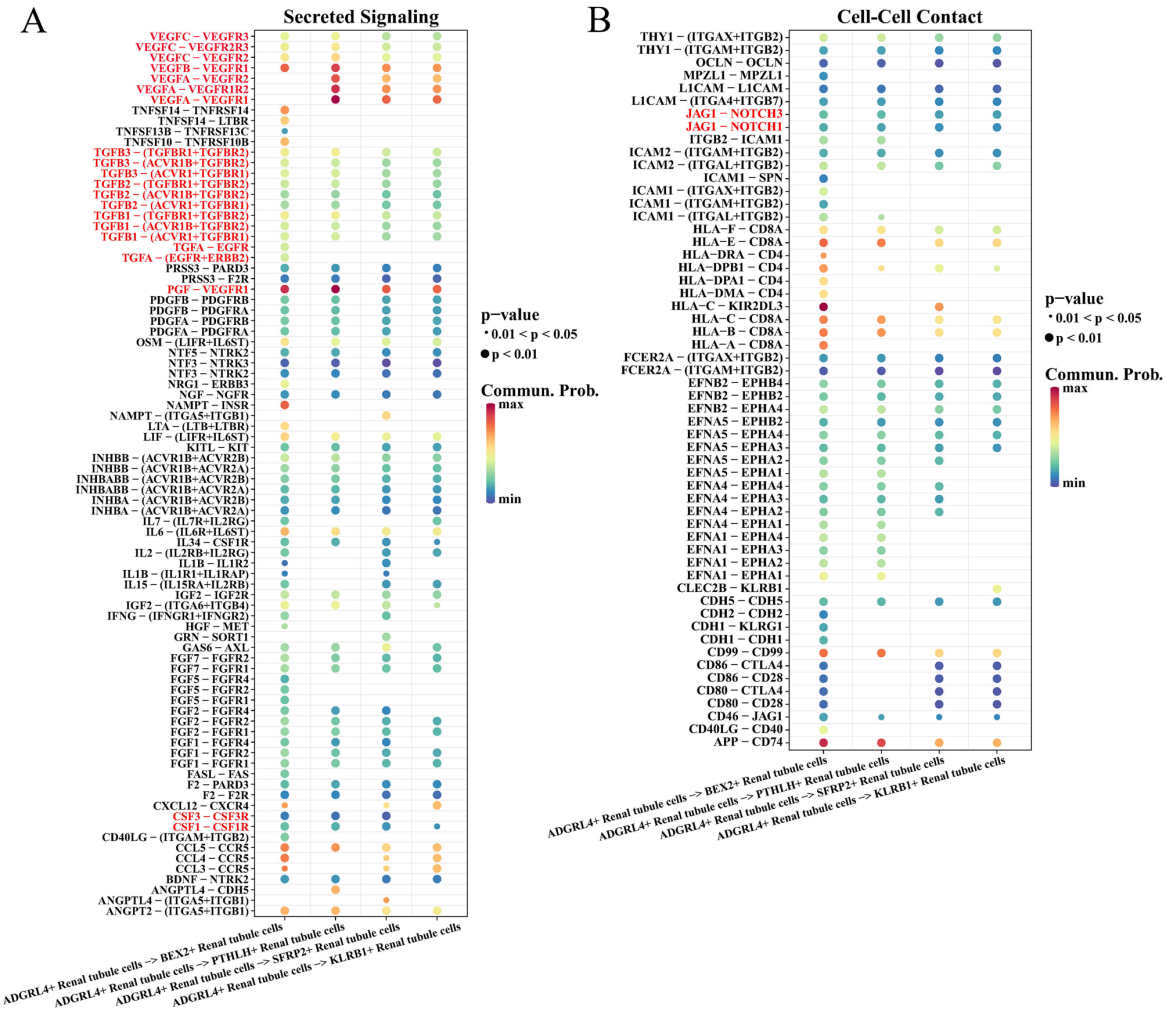
Analysis of cell communication. (**A**) For secreted signaling, bubble diagram of ligand-receptor interaction in ADGRL4+ renal tubule cells with other Renal tubule cell subclusters. (**B**) For Cell–Cell Contact, bubble diagram of ligand-receptor interaction relationship in ADGRL4+ renal tubule cells with other Renal tubule cell subclusters. The gradient from blue to red represents a change in interaction from small to large.

Ligand receptor analysis indicated that ADGRL4+ renal tubule cells might be an inducer of renal tubule cell subcluster proliferation. Specifically, in terms of secreted signaling, the vascular endothelial-derived growth factor (VEGF), transforming growth factor beta (TGFβ), and Colony Stimulating Factor (CSF) family proteins secreted by ADGRL4+ renal tubule cells were important driving factors that acted on vascular endothelial growth factor receptor (VEGFR), transforming growth factor beta receptor (TGFβR), activin a receptor type (ACVR), colony stimulating factor receptor (CSFR) family proteins of BEX2+ renal tubule cells, PTHLH+ renal tubule cells, SFRP2+ renal tubule cells, KLRB+ renal tubule cells, which all promoted angiogenesis and metastasis (Fig. 4A). In terms of Cell–Cell Contact, Jagged Canonical Notch Ligand (JAG) on the surface of ADGRL4+ renal tubule cells and Notch receptor assemblies on other renal tubule cell subclusters promoted ccRCC cell proliferation (Fig. 4B). Therefore, ADGRL4+ renal tubule cells might be an inducer of the proliferation of subclusters of renal tubule cells.

### ADGRL4+ renal tubule cells accumulated around glomeruli and influenced the prognosis of ccRCC

**Figure 5:**
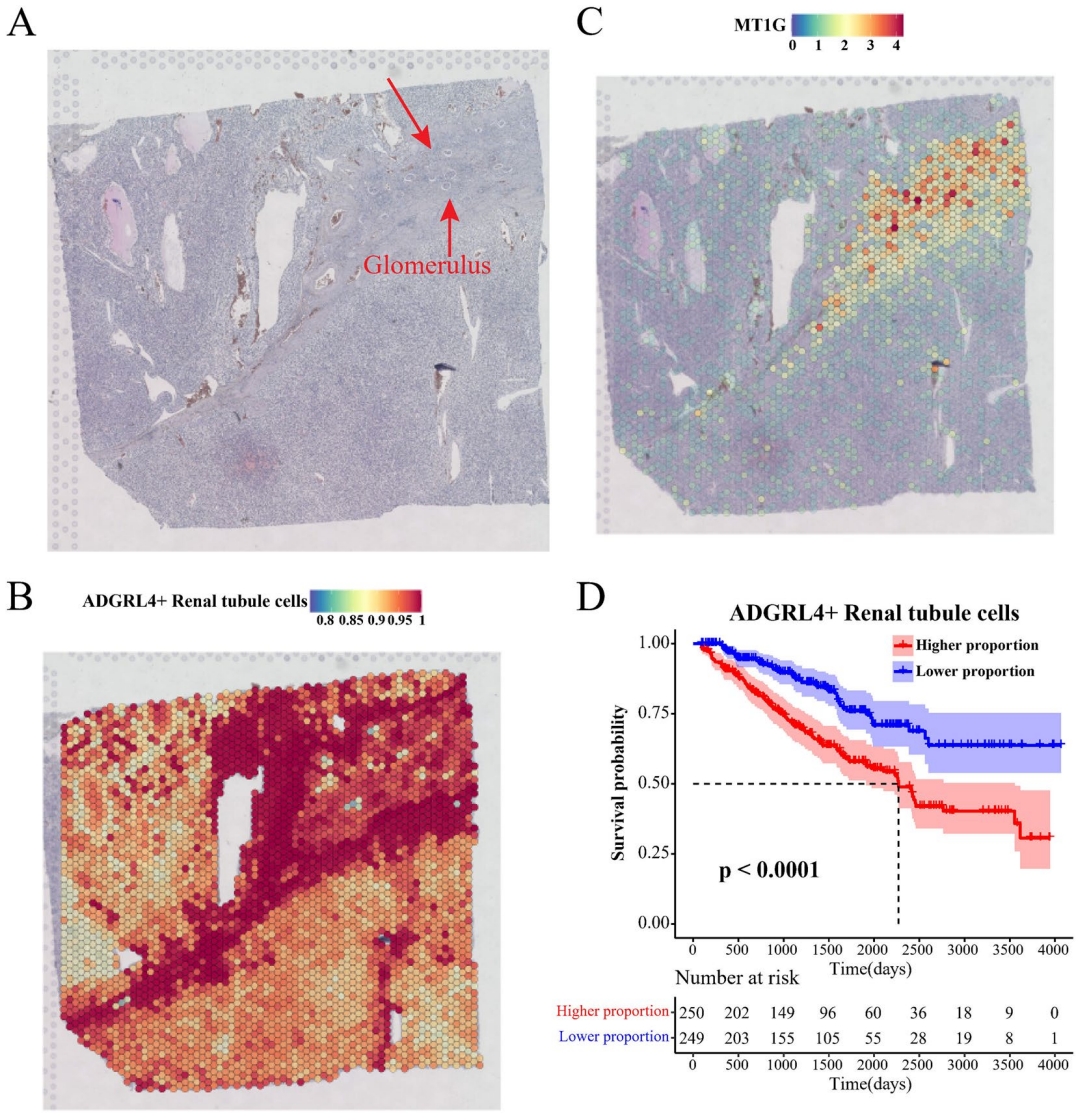
Spatial properties and prognostic implications of ADGRL4+ renal tubule cells in ccRCC. (**A**) Histological section of ccRCC tissue in GSM6415706. Glomeruli marked with arrows. (**B**) Localization of ADGRL4+ renal tubule cells in ccRCC tissues. (**C**) ADGRL4+ renal tubule cells accompanied by MT1G expression upregulation. (**D**) Survival analysis of patients with high/low ADGRL4+ renal tubule cells proportion in TCGA-KIRC.

We further evaluated the spatial properties of ccRCC tissues based on the tissue section information and spatial transcriptome data of ccRCC in GSM6415706 to delineate the spatial attributes of ADGRL4+ renal tubule cells and their effects on other renal tubule cells. Tissue section information of ccRCC from GSM6415706 was loaded (Fig. 5A). Upon mapping the spot in GSM6415706 to the sectioned tissues, we observed that ADGRL4+ renal tubule cells clustered near the glomeruli and were associated with up-regulation of MT1G expression (Fig. 5B–C). Notably, ccRCC tissues showed limited or almost no presence of BEX2 + renal tubule cells, PTHLH + renal tubule cells, SFRP2+ renal tubule cells, KLRB+ renal tubule cells, and HGF + renal tubule cells (Supplementary Fig. 3). This indicated that ADGRL4+ renal tubule cells were an essential cancer cell subcluster in ccRCC, and might be the main causative factor for aggregation or generation among other cell subclusters. Next, the impact of renal tubule cell subclusters on the prognosis of ccRCC was explored using TCGA-KIRC. We observed that patients with low proportions of ADGRL4+ renal tubule cells, HGF + renal tubule cells, and SFRP2+ renal tubule cells clearly exhibited a remarkably better survival advantage (Fig. 5D, Supplementary Fig. 4). Overall, our results further supported that ADGRL4+ renal tubule cells were the major cell type in influencing the prognosis of ccRCC and promoting tumor development.

### Knockdown of ELK3 disturbed the migration and invasion of renal cancer cells

**Figure 6:**
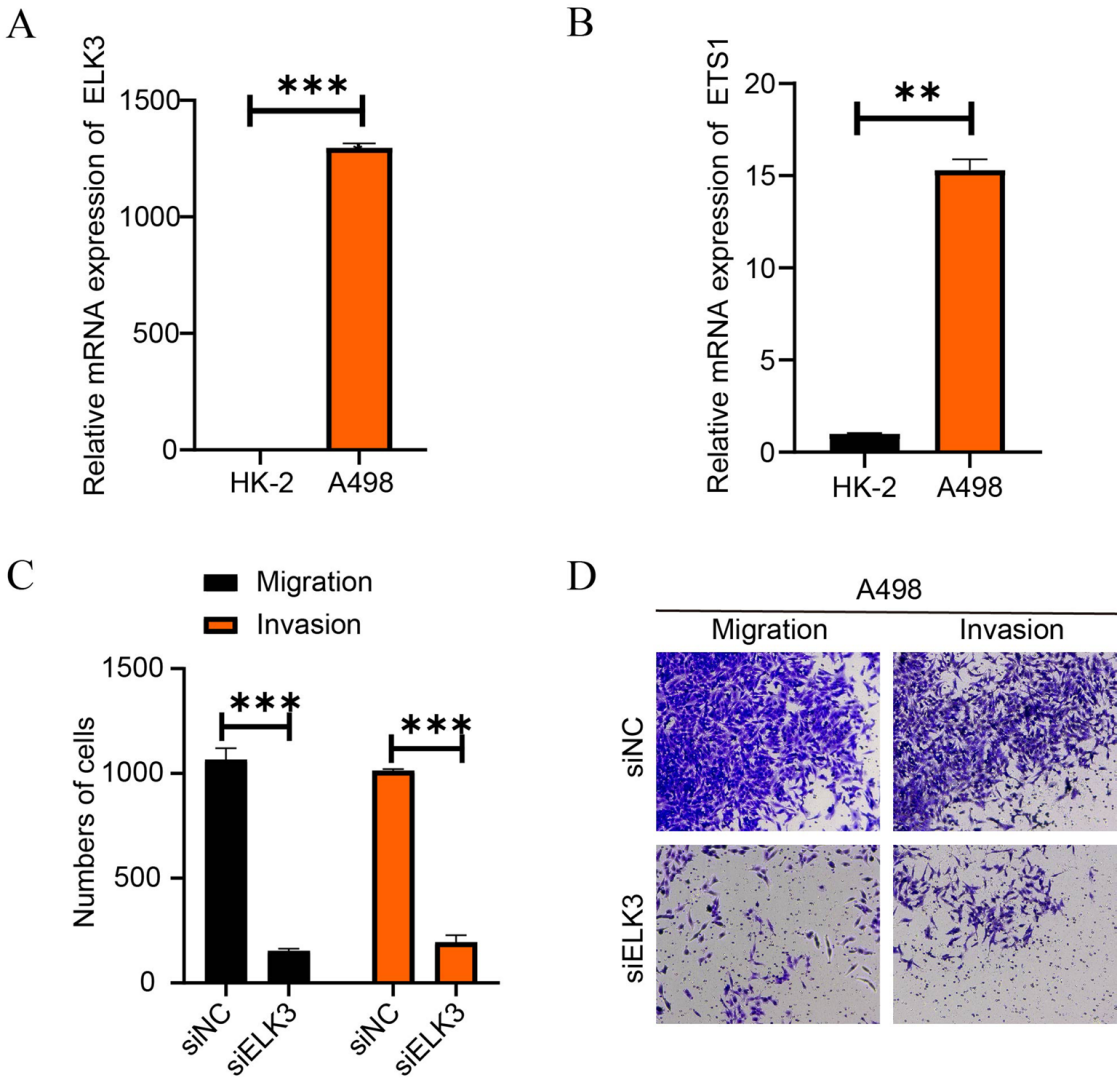
Knockdown of ELK3 disturbed the migration and invasion of renal cancer cells. (**A**) ELK3 mRNA levels in HK-2 cells and A498 cells. (**B**) ETS1 mRNA levels in HK-2 cells and A498 cells. (**C**–**D**) Migration and invasion numbers and microscopic images of A498 cells after ETS1 knockdown.

ELK3 and ETS1 mRNA levels were compared between human renal cortical proximal tubular epithelial cell line HK-2 and human renal carcinoma cell line A489. ELK3 mRNA level in A489 cells were more than 1000-fold higher than in HK-2 cells (Fig. 6A). Similarly, ETS1 mRNA levels were much higher in A489 cells than in HK-2 cells (Fig. 6B). Knockdown of ELK3 in A489 cells significantly affected the cell migration and invasion (Fig. 6C–D).

## Discussion

Tumorigenesis typically involves complex interactions with surrounding environmental factors, and different environments suggest different directions of differentiation and evolution^28^. Tumor heterogeneity refers to the phenomenon that tumor cells interact with influential factors in the tumor microenvironment, with different evolutionary directions in temporal and spatial dimensions^12^. Tumor heterogeneity also means the presence of multiple cell subpopulations of cancer cells, and the histological and cell biological behaviors or characteristics of these cell subpopulations contribute to the complexity and diversity of tumor genetics^29^. Genetic properties of diverse tumor cells caused by non-genetic factors (environmental interactions) require further study at the single-cell level^30^. CcRCC is highly heterogeneous with a complex genetic predisposition^7,8^. Characterizing critical cell populations in ccRCC tumor tissues could help to mine effective therapeutic targets.

This study characterized the single-cell landscape in ccRCC tissues and para-tumor tissues in GSE156632 based on NNMT, a high-expressed oncogene in renal tubule cells in ccRCC, and then classified six heterogeneous subclusters of renal tubule cells. ADGRL4+ renal tubule cells were associated with angiogenesis, cell migration responses. ADGRL4 is also influenced by ELTD1 (https://www.ncbi.nlm.nih.gov/gene/64123). ADGRL4/ELTD1 affecting tumor angiogenesis has been reported by several studies. Huang et al.^31^ showed that targeting ADGRL4/ ELTD1 restores vascular function and improves prognosis in gliomas. A mouse breast cancer model with recombinant ADGRL4/ELTD1 expression exhibits metastasis to lung, more tumorous tissues and larger tumor vascular size^32^. In metastatic renal cell cancer, patients with high ADGRL4/ELTD1 expression in the tumor vasculature respond better to sunitinib treatment^33^.

Furthermore, ETS1 and ELK3-dominant GRNs were remarkably activated in ADGRL4+ renal tubule cells. ETS1 induces aggressive tumor cell generation, resistance of tumor cells, and angiogenesis^34^. ETS1 is a metastasis-associated risk factor in ccRCC, specifically, downregulated ETS1 markedly inhibits cell proliferation, metastasis, and invasive capacity^35,36^, while upregulated ETS1 increases ccRCC susceptibility^37^. ELK3 is also an essential metastatic risk factor in other types of cancers^38–40^. The transcriptional activity between ETS1 and ELK3 is also a regulator of angiogenesis^41^. Thus, ADGRL4+ renal tubule cells might be an aggressive cell type in ccRCC. The results of cell experiments were performed to compare the mRNA levels of ELK3 and ETS1 between human renal cortical proximal tubular epithelial cell line HK-2 and human renal carcinoma cell line A498. Functionally, knockdown of ELK3 in A498 significantly affected the cell migration and invasion.

The cell communication analysis showed that ADGRL4+ renal tubule cells interacted intensively with other subclusters through VGEF, TGFB, and CSF. As an important angiogenesis factor in cancers, VGEF is closely related to the initial development and metastasis of cancers. Angiogenesis accelerates tumor growth, invasion, and provision of a progressive tumor microenvironment^42^. In the early stage of cancer, TGFB controls cell proliferation and apoptotic process, and when the cancer entered the advanced stage, TGFB induces angiogenesis, epithelial mesenchymal transition and tumor immune escape, ultimately leading to tumor cell invasion and metastasis to different sites^43^. Binding co-expression of CSF1/CSF1R derived from renal cell cancer enhances cancer cell growth^44^. Our spatial transcriptome results indicated that ADGRL4+ renal tubule cells were remarkably enriched around glomerular tissues and other cell clusters limitedly or rarely existed in ccRCC. This phenomenon might be caused by sampling differences. The results in TCGA-KIRC showed that the abundance of ADGRL4+ renal tubule cells and SFRP2+ renal tubule cells were prognostic factors for ccRCC, and that there was a close cell-to-cell interaction between them. Intensive cell communication in ADGRL4+ renal tubule cells might promote SFRP2+ renal tubule cells abundance and regulate prognosis of ccRCC patients, suggesting that targeting ADGRL4+ renal tubule cells might be a novel therapeutic direction for ccRCC treatment.

Some limitations in this report should be noticed. ADGRL4 was less studied in ccRCC, and we did not perform wet experiment to explore the specific function of ADGRL4, which demands detailed analysis to discuss the role of ADGRL4 in ccRCC. Overall, this study portrayed the cell landscape in ccRCC tissues based on scRNA-seq data in GSE156632. Renal tubule cells were the main cancer cell type with strong tumor heterogeneity in ccRCC. Angiogenic and metastatic signaling pathways were activated in ADGRL4+ renal tubule cells, which might be induced by the highly metastatic characteristics of ccRCC. The current findings supported that ADGRL4+ renal tubule cells potentially represented a novel therapeutic target for ccRCC treatment and prognostic prediction.

### Supplementary Information

Supplementary Information 1.

Supplementary Information 2.

Supplementary Information 3.

Supplementary Information 4.

Supplementary Information 5.

## Data Availability

The datasets generated and/or analyzed during the current study are available in the [GSE156632] repository, [https://www.ncbi.nlm.nih.gov/geo/query/acc.cgi?acc=GSE156632].

## Abbreviations

ccRCC: Clear cell renal cell carcinoma
ICB: Immune checkpoint blockade
scRNA-seq: Single-cell sequencing
GEO: Gene Expression Omnibus
UCSC: University of Cingifornia Sisha Cruz
TCGA: The Cancer Genome Atlas
KIRC: Kidney clear cell carcinoma
NNMT: Nicotinamide *N*-methyltransferase
UMAP: Uniform Manifold Approximation and Projection
KNN: K-nearest neighbor
DEGs: Differentially expressed genes
FC: Fold change
GO: Gene ontology
BP: Biological process
GRNs: Gene regulatory networks
TFs: Transcription factors
K–M: Kaplan–Meier
VEGF: Vascular endothelial-derived growth factor
TGFB: Transforming growth factor beta
CSF: Colony stimulating factor
VEGFR: Vascular endothelial growth factor receptor
TGFBR: Transforming growth factor beta receptor
ACVR: Activin a receptor type
CSFR: Colony stimulating factor receptor
JAG: Jagged canonical Notch ligand
