# Supplementary figures and images for: Single-cell analysis reveals ADGRL4+ renal tubule cells as a highly aggressive cell type in clear cell renal cell carcinoma
Source: Sci Rep. 2024 Jan 29;14:2407. doi: 10.1038/s41598-024-52928-1 (PMC10824758; doi:10.1038/s41598-024-52928-1)

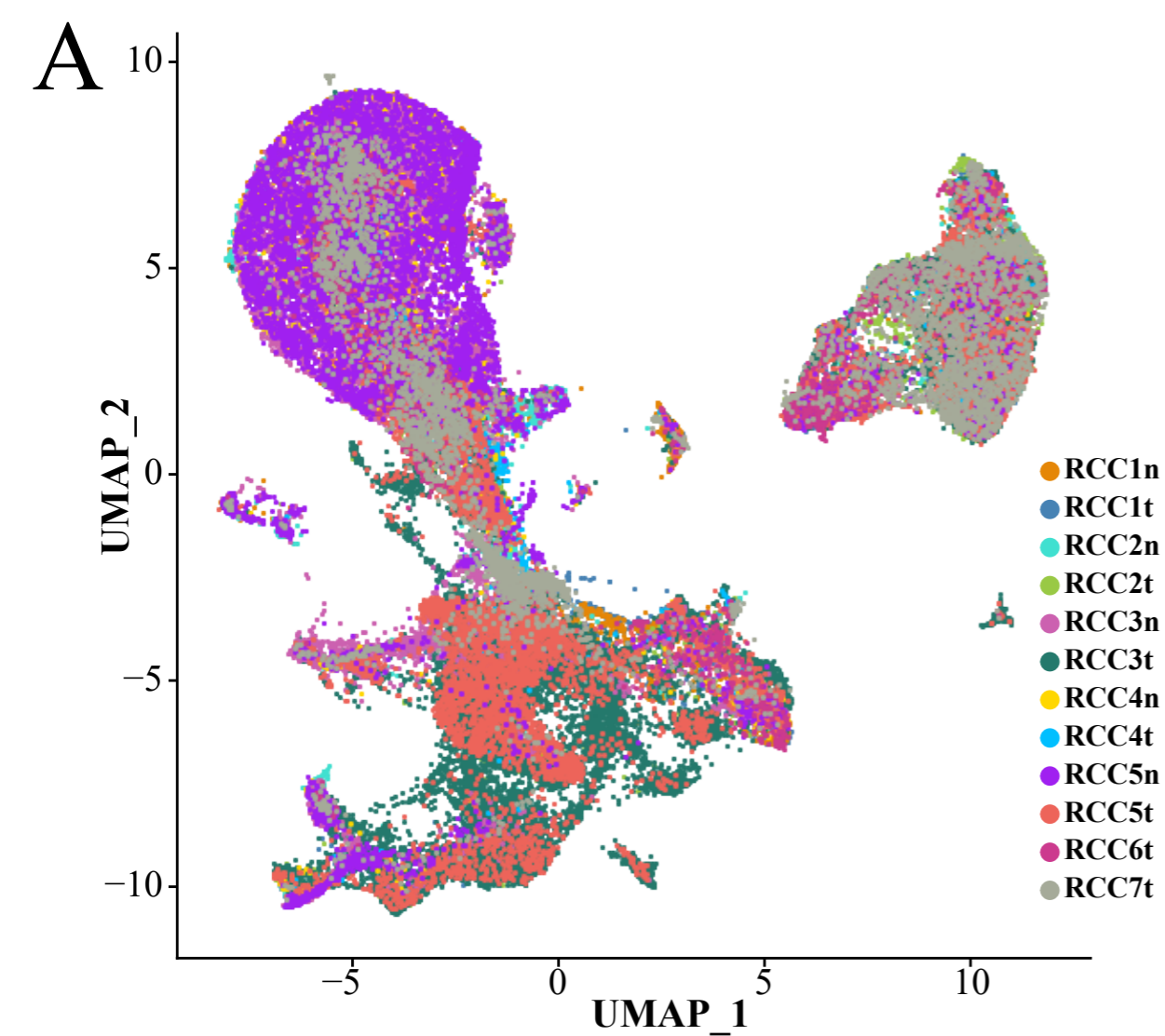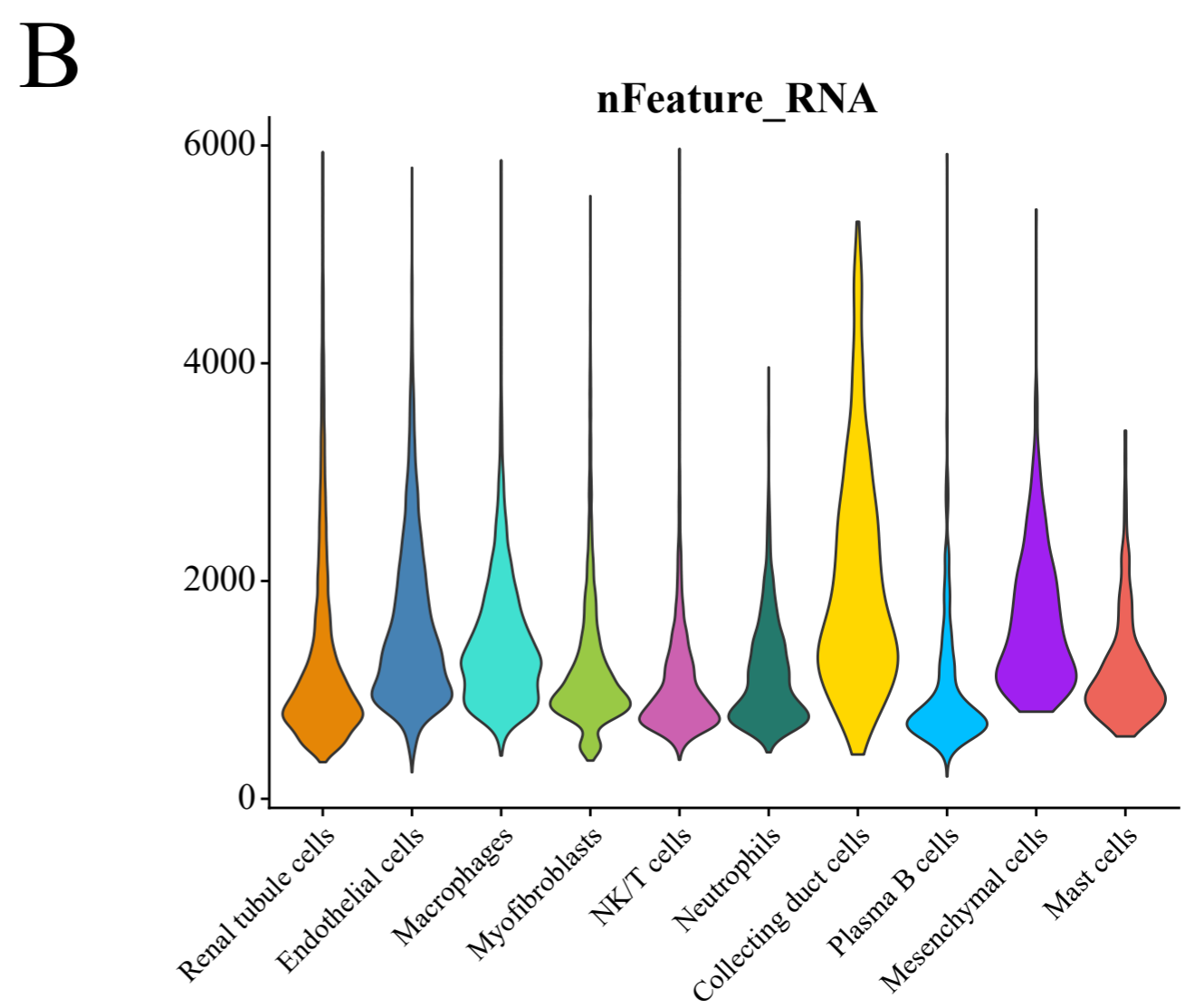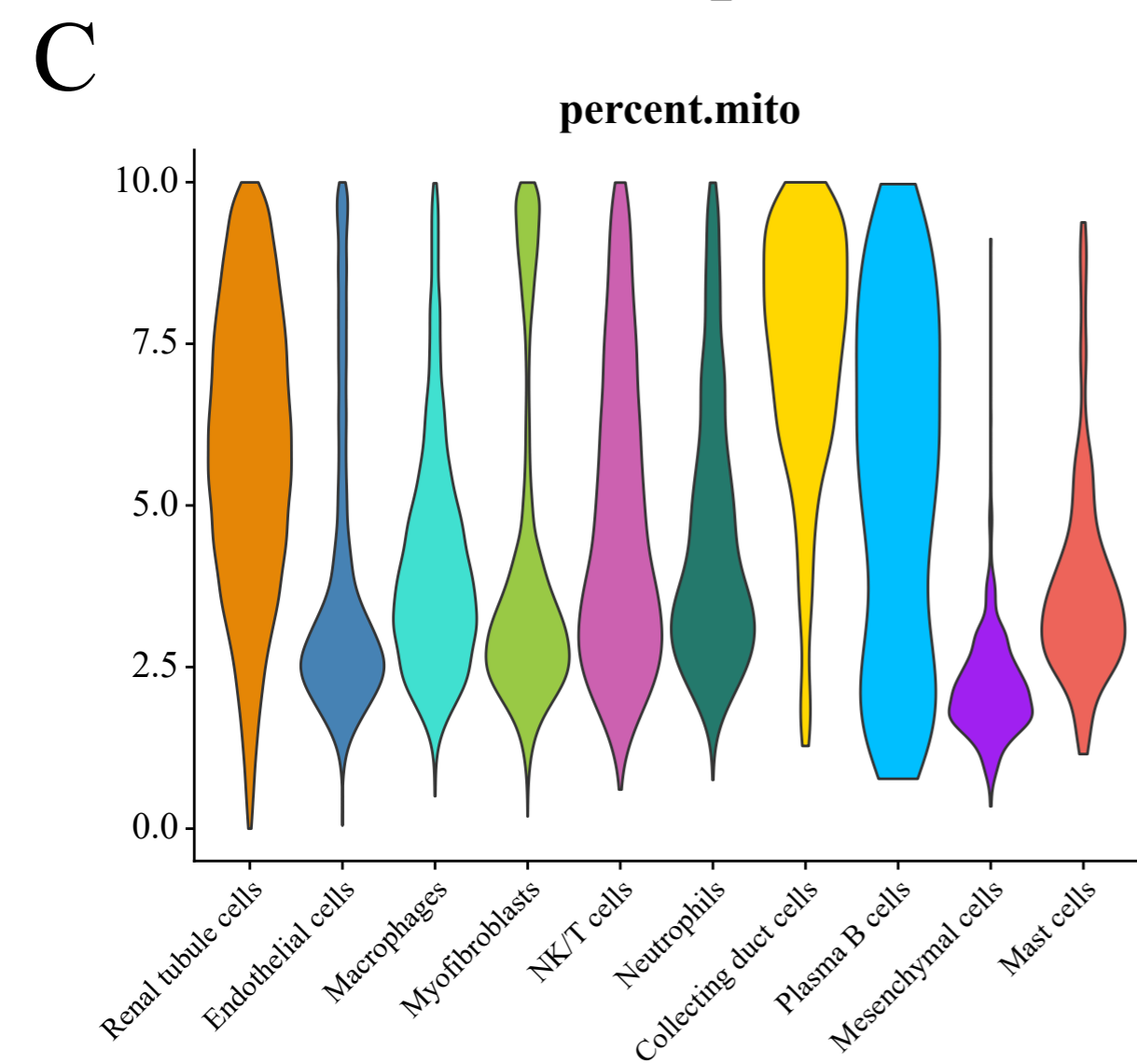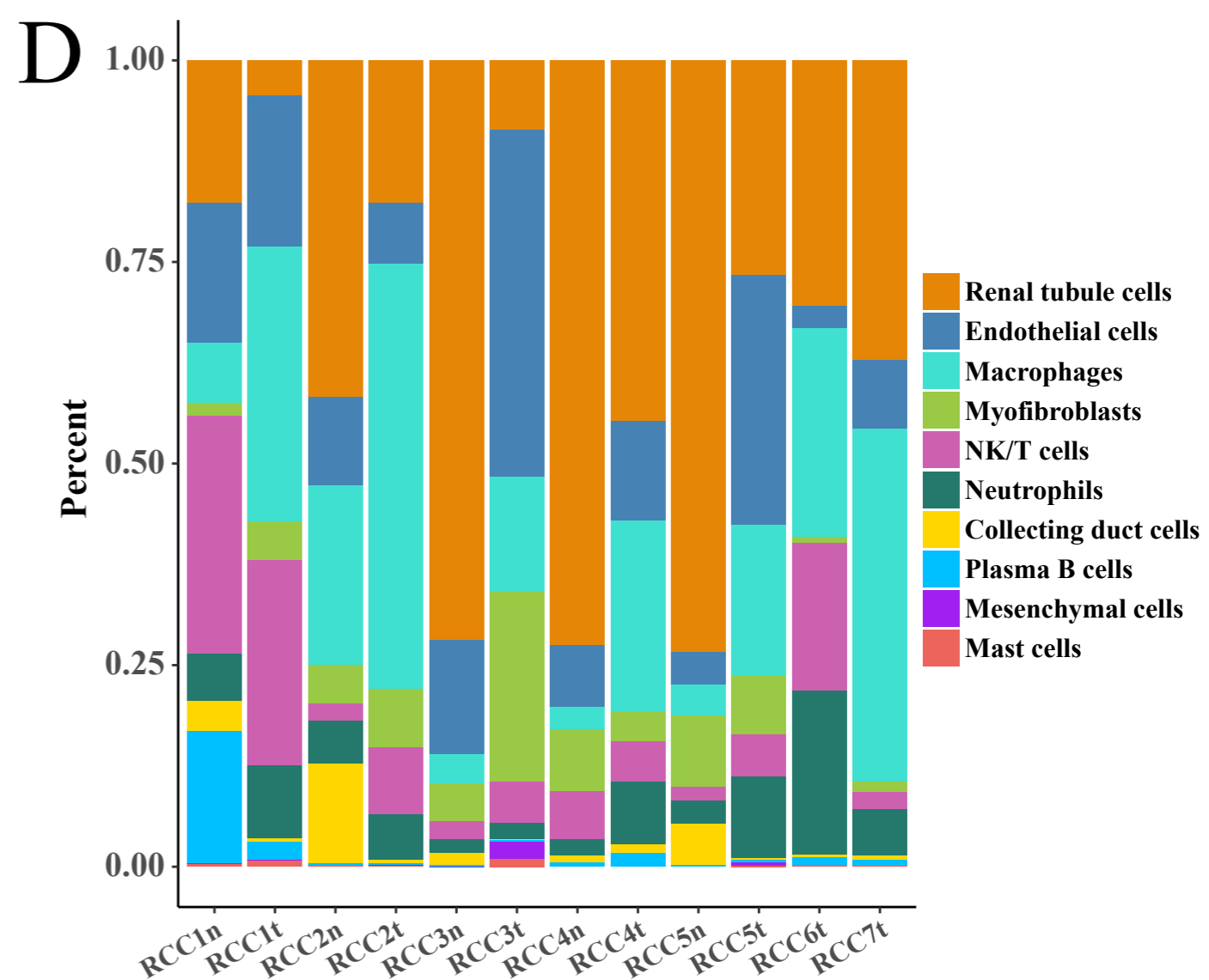

Supplement: Supplementary file 1 — Supplementary Information 1. [file 41598_2024_52928_MOESM1_ESM.pdf]

A

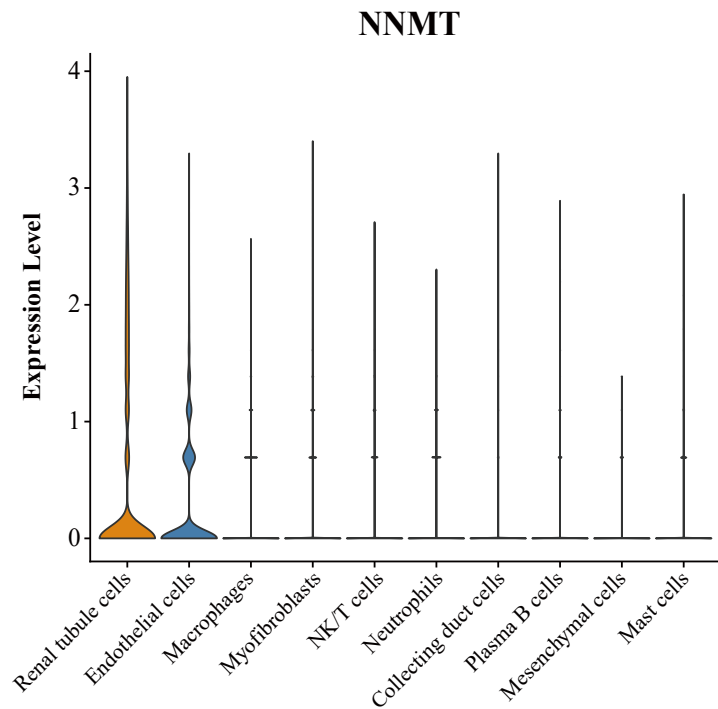

B

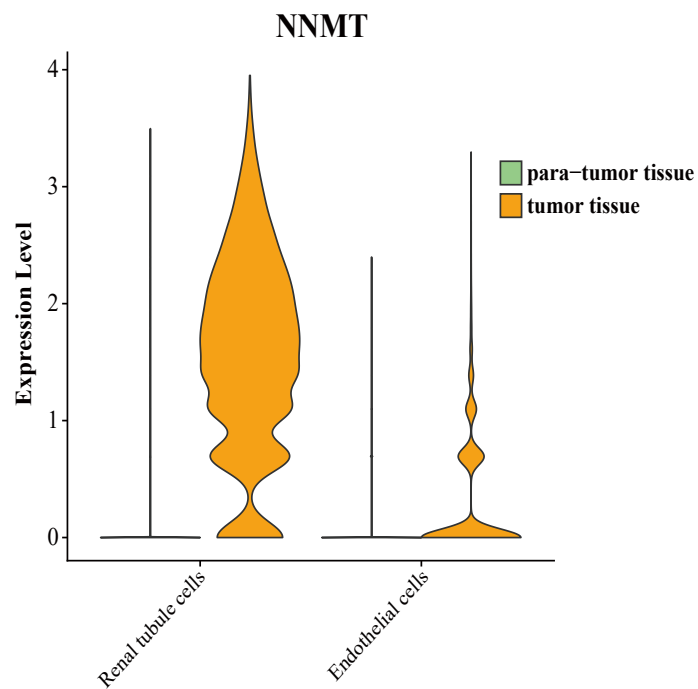

Supplement: Supplementary file 2 — Supplementary Information 2. [file 41598_2024_52928_MOESM2_ESM.pdf]
