# Supplementary material for: Single-cell analysis reveals ADGRL4+ renal tubule cells as a highly aggressive cell type in clear cell renal cell carcinoma
Source: Sci Rep. 2024 Jan 29;14:2407. doi: 10.1038/s41598-024-52928-1 (PMC10824758; doi:10.1038/s41598-024-52928-1)
Supplement: Supplementary file 3 — Supplementary Information 3. [file 41598_2024_52928_MOESM3_ESM.pdf]

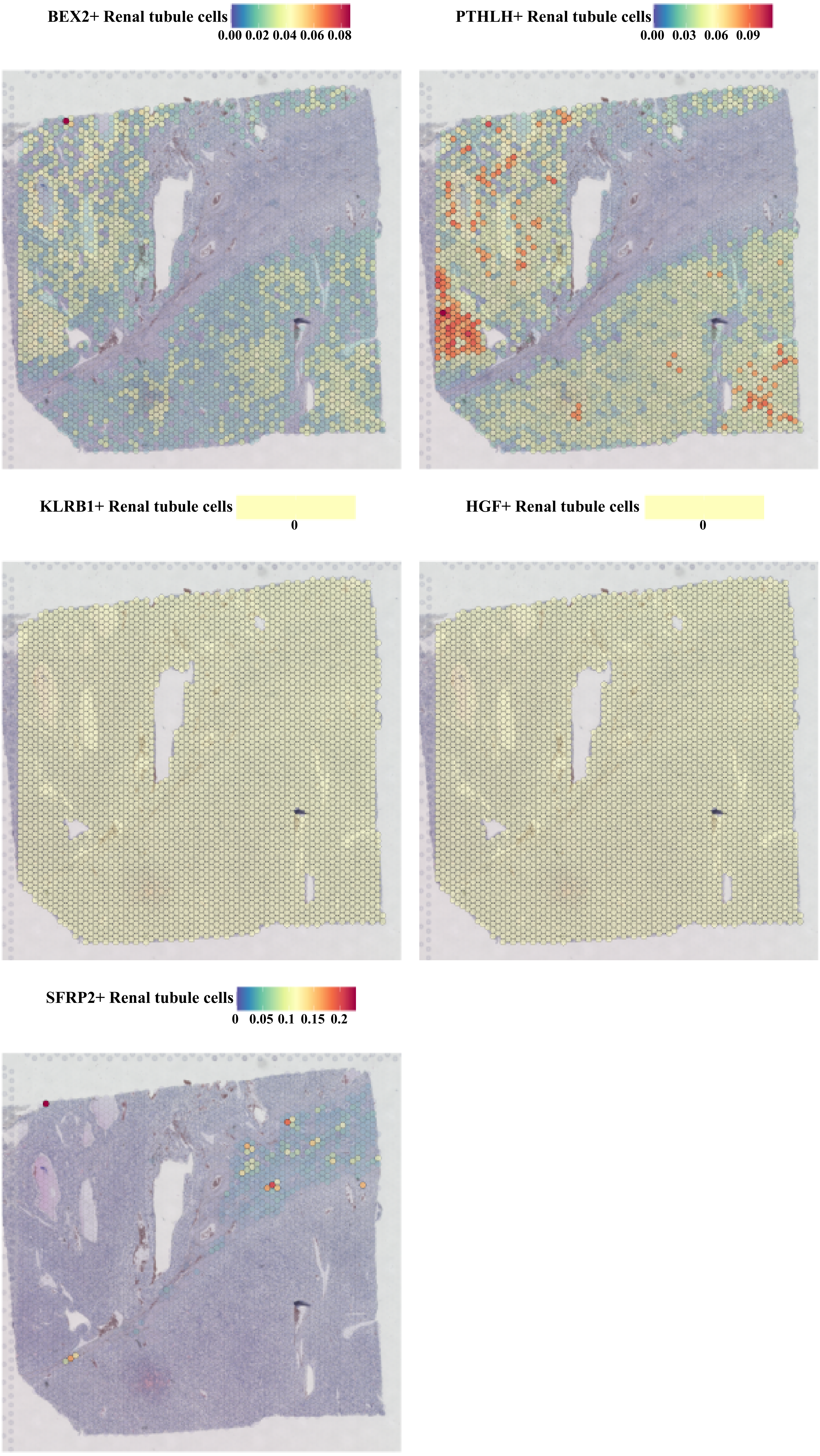
BEX2+ Renal tubule cells
0.00 0.02 0.04 0.06 0.08
PTHLH+ Renal tubule cells
0.00 0.03 0.06 0.09
KLRB1+ Renal tubule cells
0
HGF+ Renal tubule cells
0
SFRP2+ Renal tubule cells
0 0.05 0.1 0.15 0.2
