# Supplementary material for: Single-cell analysis reveals ADGRL4+ renal tubule cells as a highly aggressive cell type in clear cell renal cell carcinoma
Source: Sci Rep. 2024 Jan 29;14:2407. doi: 10.1038/s41598-024-52928-1 (PMC10824758; doi:10.1038/s41598-024-52928-1)
Supplement: Supplementary file 4 — Supplementary Information 4. [file 41598_2024_52928_MOESM4_ESM.pdf]

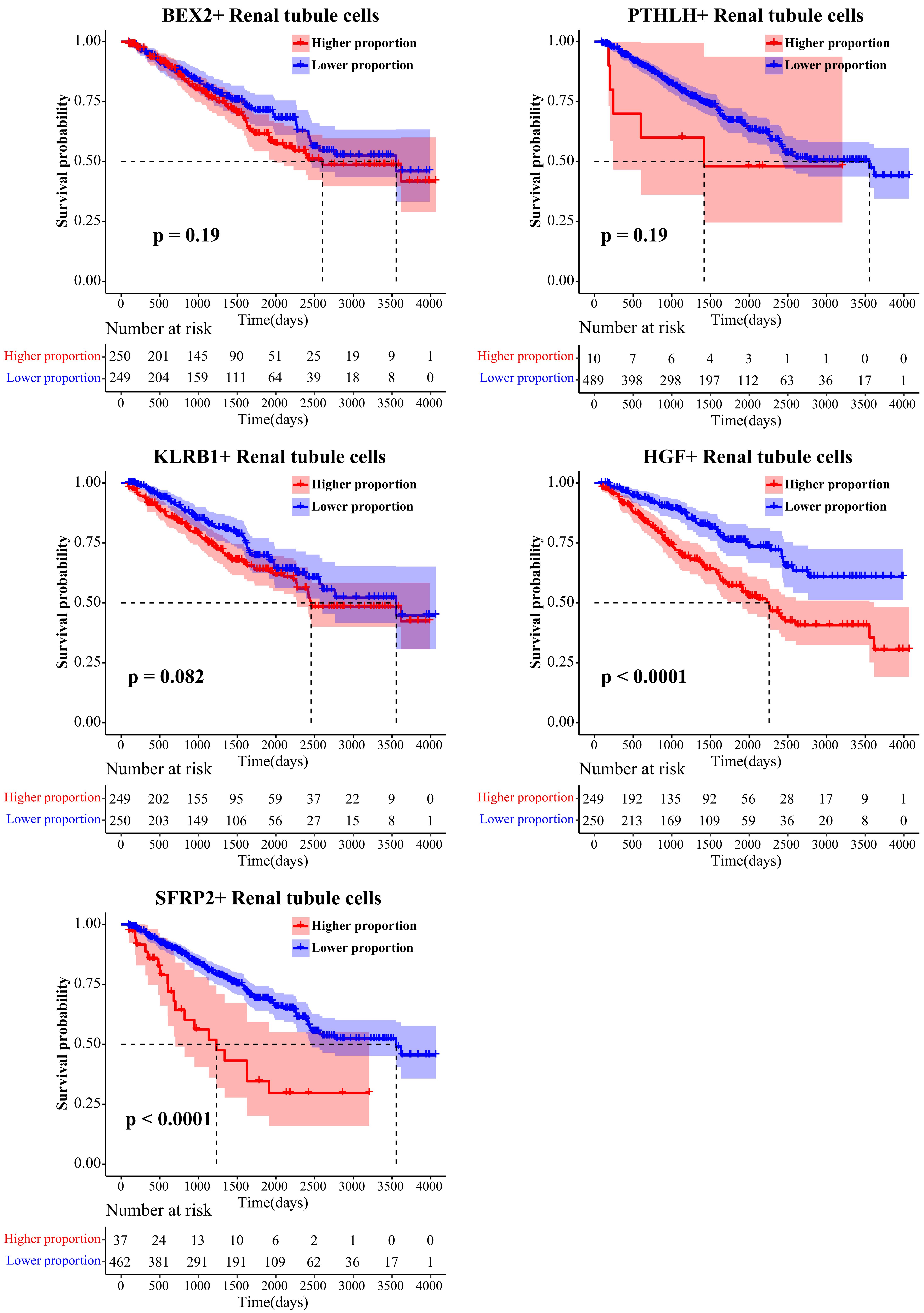
BEX2+ Renal tubule cells
Higher proportion
Lower proportion
Survival probability
1.00
0.75
0.50
0.25
0.00
p = 0.19
0 500 1000 1500 2000 2500 3000 3500 4000
Time(days)
Number at risk
Higher proportion 250 201 145 90 51 25 19 9 1
Lower proportion 249 204 159 111 64 39 18 8 0
0 500 1000 1500 2000 2500 3000 3500 4000
Time(days)
PTHLH+ Renal tubule cells
Higher proportion
Lower proportion
Survival probability
1.00
0.75
0.50
0.25
0.00
p = 0.19
0 500 1000 1500 2000 2500 3000 3500 4000
Time(days)
Number at risk
Higher proportion 10 7 6 4 3 1 1 0 0
Lower proportion 489 398 298 197 112 63 36 17 1
0 500 1000 1500 2000 2500 3000 3500 4000
Time(days)
KLRB1+ Renal tubule cells
Higher proportion
Lower proportion
Survival probability
1.00
0.75
0.50
0.25
0.00
p = 0.082
0 500 1000 1500 2000 2500 3000 3500 4000
Time(days)
Number at risk
Higher proportion 249 202 155 95 59 37 22 9 0
Lower proportion 250 203 149 106 56 27 15 8 1
0 500 1000 1500 2000 2500 3000 3500 4000
Time(days)
HGF+ Renal tubule cells
Higher proportion
Lower proportion
Survival probability
1.00
0.75
0.50
0.25
0.00
p < 0.0001
0 500 1000 1500 2000 2500 3000 3500 4000
Time(days)
Number at risk
Higher proportion 249 192 135 92 56 28 17 9 1
Lower proportion 250 213 169 109 59 36 20 8 0
0 500 1000 1500 2000 2500 3000 3500 4000
Time(days)
SFRP2+ Renal tubule cells
Higher proportion
Lower proportion
Survival probability
1.00
0.75
0.50
0.25
0.00
p < 0.0001
0 500 1000 1500 2000 2500 3000 3500 4000
Time(days)
Number at risk
Higher proportion 37 24 13 10 6 2 1 0 0
Lower proportion 462 381 291 191 109 62 36 17 1
0 500 1000 1500 2000 2500 3000 3500 4000
Time(days)
