# Supplementary material for: Single-cell analysis reveals ADGRL4+ renal tubule cells as a highly aggressive cell type in clear cell renal cell carcinoma
Source: Sci Rep. 2024 Jan 29;14:2407. doi: 10.1038/s41598-024-52928-1 (PMC10824758; doi:10.1038/s41598-024-52928-1)
Supplement: Supplementary file 5 — Supplementary Information 5. [file 41598_2024_52928_MOESM5_ESM.docx]

**Supplementary Figure 1: scRNA-seq data analysis.**

A: Cell distribution graph of samples corrected for batch effect.

B: Number of RNA_count in 10 cell types.

C: Proportion of mitochondrial genes in 10 cell types.

D: Histogram of the percentage of 10 cell types in ccRCC and para-tumor tissues.

**Supplementary Figure 2: NNMT expression levels.**

A: violin plot of NNMT expression level in 10 cell types.

B: NNMT expression levels in Renal tubule cells, Endothelial cells in ccRCC and para-tumor tissues.

**Supplementary Figure 3: Distribution and proportion of BEX2+ Renal tubule cells, PTHLH+ Renal tubule cells, KLRB1+ Renal tubule cells, HGF+ Renal tubule cells, SFRP2+ Renal tubule cells, HGF+ Renal tubule cells, SFRP2+ Renal tubule cells.**

**Supplementary Figure 4: Survival analysis of BEX2+ Renal tubule cells, PTHLH+ Renal tubule cells, KLRB1+ Renal tubule cells, HGF+ Renal tubule cells, SFRP2+ Renal tubule cells abundance groups in TCGA-KIRC.**
